# Somatostatin Analogue Treatment of a TSH-Secreting Adenoma Presenting With Accelerated Bone Metabolism and a Pericardial Effusion: A Case Report

**DOI:** 10.1097/MD.0000000000002358

**Published:** 2016-01-15

**Authors:** Athanasios C. Mousiolis, Eleni Rapti, Maria Grammatiki, Maria Yavropoulou, Maria Efstathiou, Nikolaos Foroglou, Michalis Daniilidis, Kalliopi Kotsa

**Affiliations:** From the Division of Endocrinology (ACM, ER, MG, MY, KK); Department of Neurosurgery (NF); and 1st Department of Internal Medicine (ME, MD), AHEPA Hospital, Aristotle University, Thessaloniki, Greece.

## Abstract

Increased bone turnover and other less frequent comorbidities of hyperthyroidism, such as heart failure, have only rarely been reported in association with central hyperthyroidism due to a thyrotropin (TSH)-secreting pituitary adenoma (TSHoma). Treatment is highly empirical and relies on eliminating the tumor and the hyperthyroid state.

We report here an unusual case of a 39-year-old man who was initially admitted for management of pleuritic chest pain and fever of unknown origin. Diagnostic work up confirmed pericarditis and pleural effusion both refractory to treatment. The patient had a previous history of persistently elevated levels of alkaline phosphatase (ALP), indicative of increased bone turnover. He had also initially been treated with thyroxine supplementation due to elevated TSH levels. During the diagnostic process a TSHoma was revealed. Thyroxine was discontinued, and resection of the pituitary tumor followed by treatment with a somatostatin analog led to complete recession of the effusions, normalization of ALP, and shrinkage of pituitary tumor.

Accelerated bone metabolism and pericardial and pleural effusions attributed to a TSHoma may resolve after successful treatment of the tumor. The unexpected clinical course of this case highlights the need for careful long-term surveillance in patients with these rare pituitary adenomas.

## INTRODUCTION

Central hyperthyroidism due to a thyrotropin (TSH)-secreting pituitary adenoma (TSHoma) is a rare cause of hyperthyroidism. Clinical signs and symptoms of hyperthyroidism due to a TSHoma are usually milder than in primary hyperthyroidism, and the disease may remain undiagnosed until tumor expansion signs develop.^1–3^ Effusions (pericardial or pleural) are caused by several pathologies, infectious, neoplastic, metabolic, or traumatic. Pericardial effusion is a common manifestation of severe hypothyroidism, although it has only rarely been reported in association with hyperthyroidism.^4–7^ On the contrary, increased bone turnover is a common finding in thyrotoxicosis making hyperthyroidism one of the main causes of secondary osteoporosis. However, there are only a few reported cases of accelerated bone metabolism in association with hyperthyroidism due to a TSHoma.^8–10^ Here, we present a rare case of a 39-year-old man with a TSHoma, pericardial and pleural effusions and osteopenia with elevated alkaline phosphatase (ALP) levels. Partial resection of the pituitary tumor by trans-sphenoidal neurosurgery followed by treatment with a somatostatin analog led to complete recession of the effusions, normalization of bone turnover, and shrinkage of the pituitary tumor.

## CASE REPORT

A 39-year-old man with no previous medical history except a reported mild hypothyroidism treated with thyroxine supplementation presented to the hospital with persistent signs of dyspnea, fatigue, chest pain, and fever starting a few days earlier, without clinical improvement despite antibiotic therapy.

**FIGURE 1:**
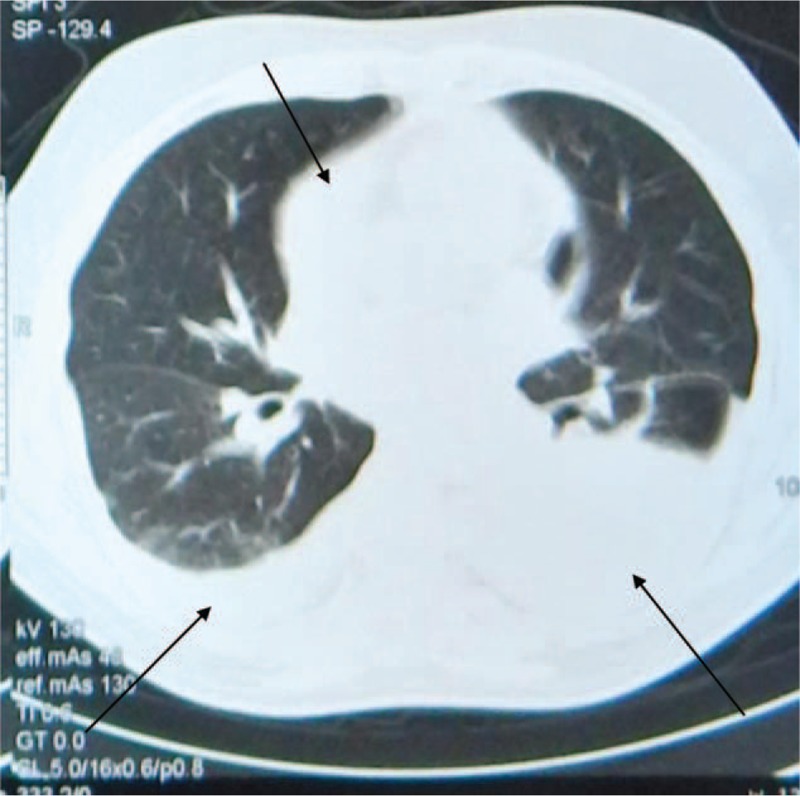
Chest-computed tomography. Pericardial and pleural effusions marked by arrows.

After a comprehensive diagnostic work up, the diagnosis of pericardial and pleural effusion of no apparent cause was made. Except leukocytosis, increased erythrocyte sedimentation rate, and C-reactive protein, all other laboratory studies, including autoimmune markers, were within normal limits (Table 1). An enlarged cardiac silhouette appeared in the chest x-ray with signs of pleural effusion, and the echocardiogram revealed a constrictive pericardial effusion without evidence of cardiac tamponade. A chest computed tomography confirmed the diagnosis (Figure 1). Pleural effusion aspiration was carried out and fluid was identified as exudative and free of cancer cells. During his hospitalization the patient received a wide spectrum of antibiotics, corticosteroids, anti-inflammatory drugs, and colchicine. Eventually, the pericardial effusion was treated surgically, but recurrence developed a few months after initial treatment.

**TABLE 1:**
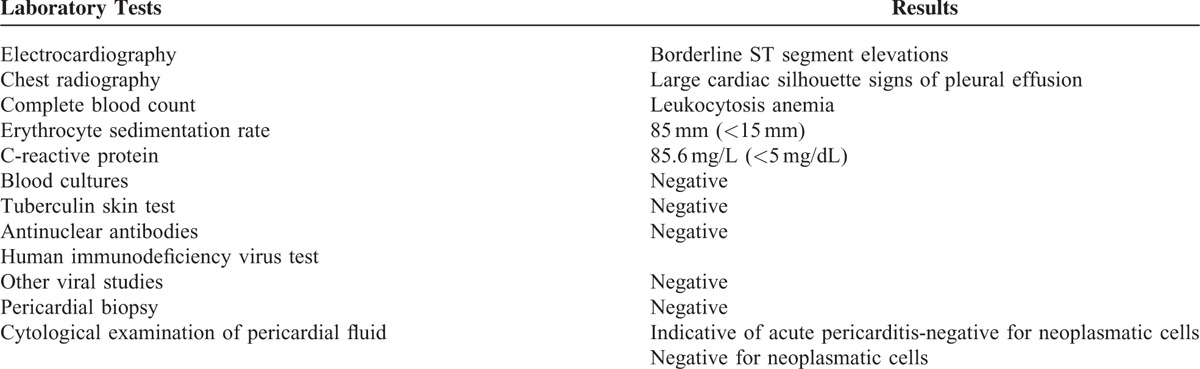
Diagnostic Evaluation of Pericarditis

The patient had been treated for years for presumed primary hypothyroidism with thyroxine supplementation based on elevated TSH levels (Table 2). During that time period there was a persistent elevation of ALP and associated osteopenia that had been attributed to vitamin D deficiency, although vitamin D supplementation was of no benefit as indicated by dual-energy x-ray absiorptiometry (DEXA L1-L4: T-score = −1.72, Z-score = −1.58). Thyroxine was discontinued, and the diagnosis of a TSHoma was suspected by persistently elevated levels of TSH accompanied by high levels of free triiodothyronine (FT3) and free thyroxine (FT4). The diagnosis was supported by classical biochemical features: an elevated a-glycoprotein subunit and a molar ratio ([a-subunit/TSH] ×10) >1 which were measured a few months after thyroxine discontinuation (Table 3).

**TABLE 2:**
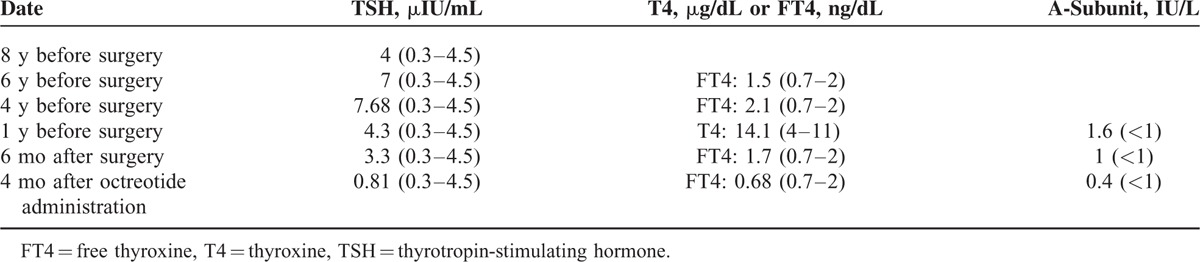
Thyroid Function Tests Pre- and Postoperatively

**TABLE 3:**
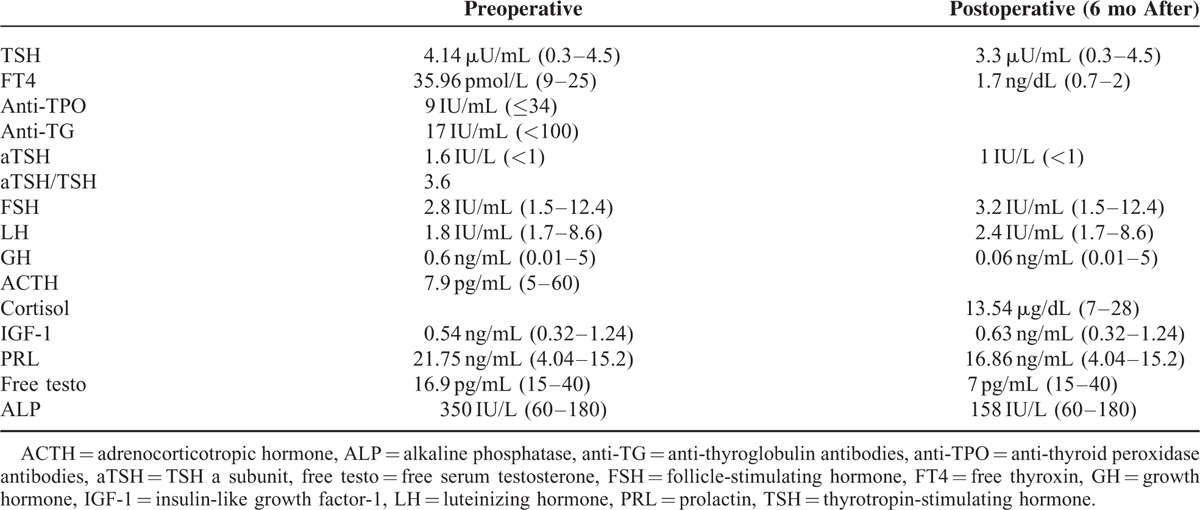
Hormone Levels and Thyroid Antibodies When TSH-Secreting Adenoma Was Diagnosed and 6 Months After Surgery

**FIGURE 2:**
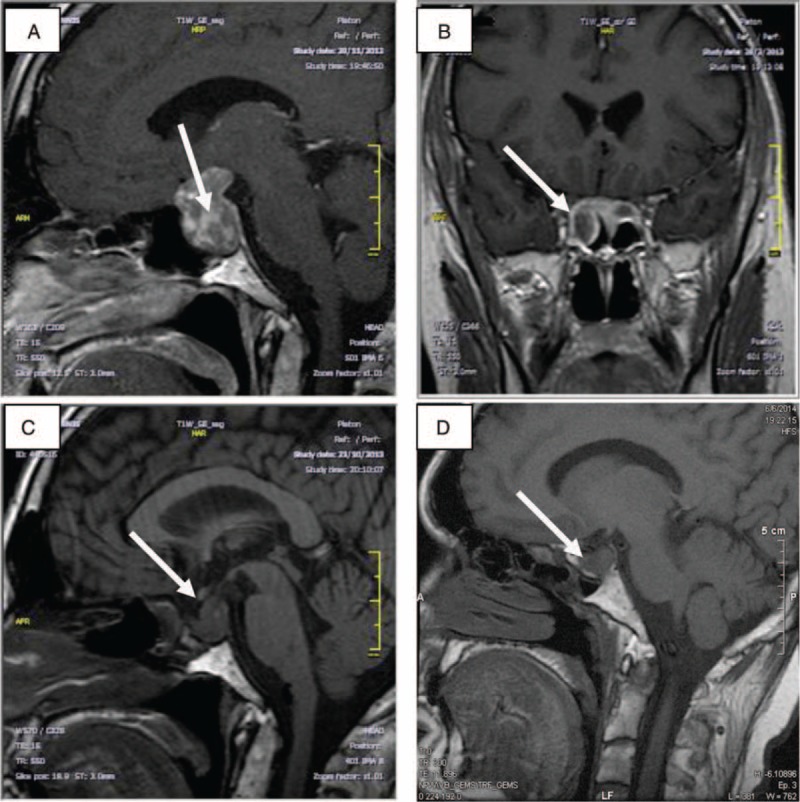
TSHoma pre- and postoperatively and 1 year after long acting octreotide treatment initiation marked by arrows: (A, B) pituitary MRI preoperatively; (C) pituitary MRI postoperatively before long acting octreotide treatment initiation; and (D) Pituitary MRI 1 year after long acting octreotide treatment initiation. MRI = magnetic resonance imaging.

Thyroid ultrasound showed a thyroid gland of normal size with heterogeneous echogenicity and a 17 mm solitary nodule with benign imaging characteristics located at the right lobe. Fine-needle aspiration biopsy was negative for malignancy. Magnetic resonance imaging revealed a pituitary macroadenoma (21 × 20 mm) with invasion of the right cavernous sinus (Figure 2). The patient had no symptoms of visual impairment or headache and his visual field test was normal.

**FIGURE 3:**
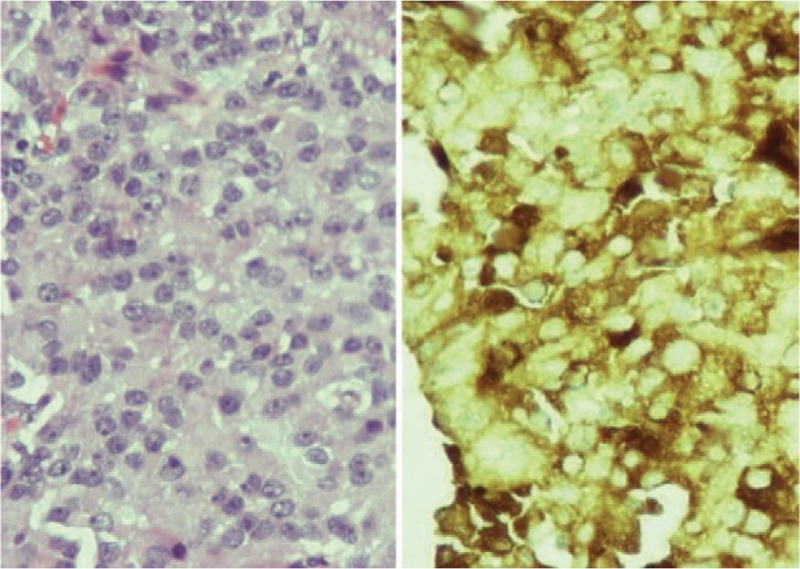
Positive immunostaining for TSH pituitary secreting adenoma (×100). TSH = thyrotropin.

The tumor was removed by trans-sphenoidal resection and histological examination and immunostaining confirmed the presence of TSH producing cells (Figure 3).

**FIGURE 4:**
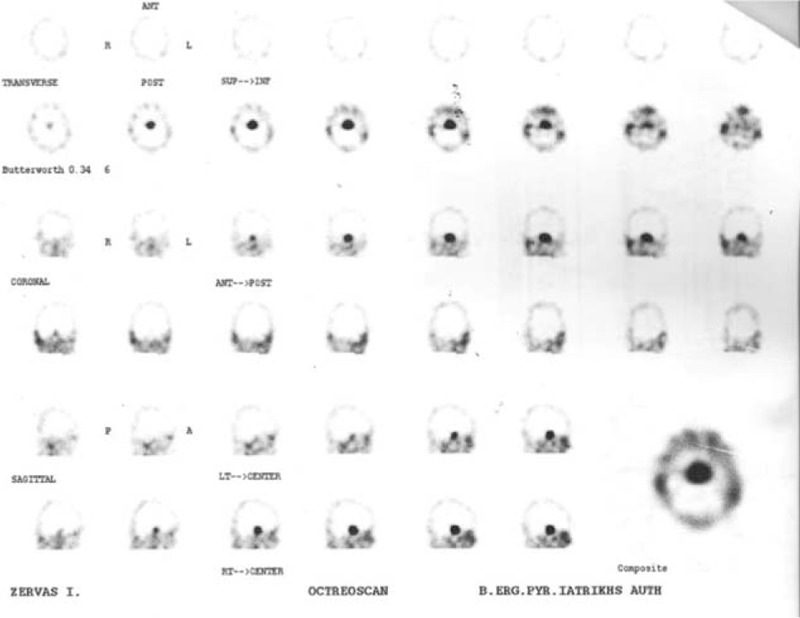
Uptake of indium-111-DTPA-octreotide postoperatively.

After surgery, due to incomplete resection (Figure 2) and persistent—although mild—elevation of a-glycoprotein subunit, an octreoscan was performed. As octreoscan was positive (Figure 4), treatment with long acting octreotide (OCT LAR) was initiated, to which the patient had a rapid antisecretory response. Shortly after initiation of medical treatment, a recession of the pericardial and pleural effusion was noted and ALP levels were normalized (Table 3).

Six months after OCT LAR treatment initiation, levothyroxine supplementation was added due to suppressed TSH levels accompanied by suppressed levels of FT4 and FT3. Euthyroidism was established 3 months afterward. Hypogonadotrophic hypogonadism indicated by low testosterone levels accompanied by low gonadotrophins (Table 3) prompted testosterone supplementation. One year after OCT LAR treatment there was a significant shrinkage in tumor size (Figure 2), no recurrence of pericardial or pleural effusions and an improvement in bone mineral density measurement (DEXA L1-L4: T-score = −1, 5, Z-score = −1).

## DISCUSSION

Hereby we present a unique case of a 39-year-old man with a TSHoma who presented with symptoms and signs of acute pericardial and pleural effusions and had for years an accelerated bone turnover due to central hyperthyroidism aggravated by thyroxine supplementation. OCT LAR treatment resulted in a rapid antisecretory and antitumor response and reversed all concomitant clinical and biochemical pathologies.

It is well known that the incidence of hyperthyroidism due to a TSHoma is extremely low, ranging between 1% and 2% of all cases of hyperthyroidism, and often remains undetectable.^1–3,11^ Most of the tumors are macroadenomas usually presenting with symptoms of tumor expansion such as headache and/or visual field impairment while the symptoms of hyperthyroidism seem to be milder compared with those caused by primary hyperthyroidism.

It is remarkable that only a few cases of hyperthyroidism in conjunction with heart disease have been reported and usually involve Graves’ disease with atrial fibrillation, tachycardia-induced cardiomyopathy, and congestive heart failure.^4–7, 12–15^ Thyroid hormones act on the cardiovascular system in various ways. Triiodothyronine acts directly on cardiac muscle fibers increasing the stroke volume and on cardiac pacemakers causing tachycardia that may result in atrial fibrillation. Concomitant-reduced peripheral resistance in hyperthyroid state contributes to an increase in cardiac output and venous return. The increased blood volume and the shortened ventricular filling seem to be the main causes for the high prevalence of congestive heart failure in hyperthyroidism.^14^ To our knowledge there are only 2 cases that report central hyperthyroidism with congestive heart failure.^13,14^ Pericardial effusion etiology does not commonly include hyperthyroidism as has been shown in a 2003 investigation among 204 patients with pericardial effusion that resulted in a definitive diagnosis in 107 (52.4%), with no thyrotoxicosis in any of them.^15^ However, pericardial effusion as an outcome of hyperthyroidism mostly due to Graves’ disease has occasionally been described, with the specific pathophysiological link between the 2 conditions still needs to be identified. Recent studies suggest that autoimmunity and/or the use of propylthiouracil are possible causes for the presence of pericarditis with hyperthyroidism.^16,17^ Pericardial effusion may be a very rare complication of other causes of thyrotoxicosis, as hashitoxicosis or toxic multinodular goiter.^18,19^

In these rare cases, treatment of the thyrotoxicosis paralleled the regression of the effusion pointing at an etiologic relation that has not been clarified. In our case, the patient had negative autoantibodies, did not receive propylthiouracil, and suffered from central hyperthyroidism. No case involving central hyperthyroidism with pericarditis and pleural effusion has been reported so far. Therefore, the etiologic relation, if any, was presumed to be with the thyrotoxic condition that regressed after surgery and octreotide treatment.

The use of somatostatin analogs has been authorized for the treatment of TSHomas pre- and postoperatively.^20–22^ In our case, OCT LAR was used postoperatively due to partial tumor resection and octreoscan positivity. The response was excellent, with rapid and significant tumor shrinkage and without any side effects.

An interesting evolution was the positive effect of OCT LAR therapy on the recession of pericardial and pleural effusions. A number of studies suggest that somatostatin analogs have significant immunomodulatory properties with anti-inflammatory effects in vivo, associated with suppression of proinflammatory cytokines and neuropeptides.^23,24^ A possible immunosuppressive reaction through somatostatin subtype 1 and 2 receptors in human macrophages has been proposed.^25^ Moreover, a case report has been described where OCT LAR was administered to a patient with pleural effusion of unknown etiology, who had not responded to the application of tube drainage for 20 days.^26^ After somatostatin analog administration, the amount of the drained fluid showed a marked reduction after 48 hours of treatment with complete recovery of the symptoms on the 5th day of treatment. In our case there was a complete recession of pericardial and pleural effusion postoperatively and after OCT LAR initiation and our patient remained free of symptoms.

The high preoperative ALP concentrations of our patient are attributed to his hyperthyroid state. This is supported by the fact that ALP levels normalized 6 months after surgery (Table 3). The effect of thyrotoxicosis on bone turnover has been described in histomorphometric studies that have demonstrated increased osteoclastic and osteoblastic activity.^27^ Although in hyperthyroid state both of them are increased, it is the osteoclastic activity that predominates. Thus, bone resorption is favored leading to a negative calcium balance and bone loss.^28,29^ In addition, serum levels of bone turnover markers, such as ALP and osteocalcin, are elevated and could remain high for a few months after treatment of hyperthyroidism and normalization of serum thyroid hormones.^30^ In our case, concentrations of ALP remained high for several years due to undiagnosed central hyperthyroidism. As a result of increased bone turnover, our patient developed osteopenia. Establishment of euthyroid state postoperatively and after OCT LAR initiation led to normalization of ALP levels.

## CONCLUSION

Accelerated bone turnover due to a hyperthyroid state induced by a TSHoma may be anticipated. However, effusions (pericardial, pleural) have only rarely been reported in combination with hyperthyroidism. To our knowledge, this is the first case of a TSHoma accompanied by increased bone turnover and pericardial/pleural effusion that both resolved after surgery and OCT LAR treatment of the adenoma.
